# Assessing the implementation effectiveness and safety of 1% tenofovir gel provision through family planning services in KwaZulu-Natal, South Africa: study protocol for an open-label randomized controlled trial

**DOI:** 10.1186/1745-6215-15-496

**Published:** 2014-12-19

**Authors:** Leila E Mansoor, Quarraisha Abdool Karim, Kathryn T Mngadi, Sarah Dlamini, Carl Montague, Nelisiwe Nkomonde, Nomzamo Mvandaba, Cheryl Baxter, Tanuja N Gengiah, Natasha Samsunder, Halima Dawood, Anneke Grobler, Janet A Frohlich, Salim S Abdool Karim

**Affiliations:** Centre for the AIDS Programme of Research in South Africa (CAPRISA), Durban, South Africa; Department of Epidemiology, Mailman School of Public Health, Columbia University, New York, USA; University of KwaZulu-Natal, Durban, South Africa

**Keywords:** South Africa, HIV prevention, Microbicide, Tenofovir gel, Health systems strengthening, Implementation

## Abstract

**Background:**

The Centre for the AIDS Programme of Research in South Africa (CAPRISA) 004 trial demonstrated a 39% reduction in HIV infection, with a 54% HIV reduction in women who used tenofovir gel consistently. A confirmatory trial is expected to report results in early 2015. In the interim, we have a unique window of opportunity to prepare for and devise effective strategies for the future policy and programmatic scale-up of tenofovir gel provision. One approach is to integrate tenofovir gel provision into family planning (FP) services. The CAPRISA 008 implementation trial provides an opportunity to provide post-trial access to tenofovir gel while generating empiric evidence to assess whether integrating tenofovir gel provision into routine FP services can achieve similar levels of adherence as the CAPRISA 004 trial.

**Methods/design:**

This is a two-arm, open-label, randomized controlled non-inferiority trial. A maximum of 700 sexually active, HIV-uninfected women aged 18 years and older who previously participated in an antiretroviral prevention study will be enrolled from an urban and rural site in KwaZulu-Natal, South Africa. The anticipated study duration is 30 months, with active accrual requiring approximately 12 months (following which an open cohort will be maintained) and follow-up continuing for approximately 18 months. At each of the two sites, eligible participants will be randomly assigned to receive tenofovir gel through either FP services (intervention arm) or through the CAPRISA research clinics (control arm). As part of the study intervention, a quality improvement approach will be used to assist the FP services to expand their current services to include tenofovir gel provision.

**Discussion:**

This protocol aims to address an important implementation question on whether FP services are able to effectively incorporate tenofovir gel provision for this at-risk group of women in South Africa. Provision of tenofovir gel to the women from the CAPRISA 004 trial meets the ethical obligation for post-trial access, and helps identify a potential avenue for future scale-up of microbicides within the public health system of South Africa.

**Trial registration:**

This trial was registered with the South Africa Department of Health (reference: DOH-27-0812-4129) and ClinicalTrials.gov (reference: NCT01691768) on 05 July 2012.

## Background

The Joint United Nations Program on HIV/AIDS and the World Health Organization estimate that nearly half of the 33.4 million people living with HIV/AIDS in the world are women [1]. In sub-Saharan Africa, women account for 59% of all infected adults, and HIV-positive women aged 15 to 24 years outnumber their male peers by three to one, representing 76% of the total cases in that age group [2, 3]. These figures highlight the vulnerability of women, particularly young women, to becoming infected with HIV in sub-Saharan Africa.

In addition to biological factors [4–6] that make women more vulnerable than men to acquiring HIV during sex, certain sexual behavior patterns place young women at high risk, including partnering with older men who are more likely to be infected [7], multiple concurrent relationships [8], low marriage rates [9], low consistent condom use rates [10, 11], and limited skills in negotiating safer sex practices. Gender-based violence increases vulnerability [12], and poverty increases reliance on transactional sex for survival [13]. Women are often unable to convince their male partners, especially husbands and regular partners, to use condoms or to practice monogamy. Despite the greater vulnerability of women, they have few options to reduce the transmission and acquisition of HIV. New technologies to prevent the sexual transmission of HIV in women, such as topical microbicides, are urgently needed.

Topical microbicides, an HIV prevention strategy that women can initiate or control, were first proposed more than two decades ago [14]. Since then several candidate microbicides have entered effectiveness trials to assess their impact on the prevention of HIV infection, including surfactants that disrupt cell membranes [15–18], polyanions that prevent attachment to target cells in the vagina [19–21], and products that maintain low vaginal pH in the presence of ejaculate [22]. Most of the candidate microbicides that have been tested in late stage prevention trials have not shown protection against HIV infection [15–17, 20, 23, 24], and some products were even potentially harmful [18, 19, 23].

Currently, research on microbicides is focused on assessing potential antiretroviral agents in various formulations and dosing strategies for their ability to prevent HIV infection. Tenofovir, a nucleotide analog with potent activity against retroviruses, is the candidate in the most advanced stage of testing. It was originally formulated for oral use as tenofovir disoproxil fumarate (TDF) [25] and its efficacy in suppressing viral replication, favorable safety profile, long half-life [26] and accessibility made it an ideal choice to be formulated into a microbicide gel.

In the Centre for the AIDS Programme of Research in South Africa (CAPRISA) 004 trial, tenofovir gel was shown to be safe [27] and provided an overall protective effect of 39% against HIV infection and 51% against herpes simplex virus type 2 (HSV-2) infection among women in KwaZulu-Natal, South Africa [28, 29].

Although the results of the CAPRISA 004 trial still need to be confirmed, tenofovir gel could potentially fill an important HIV prevention gap, especially for women unable to successfully negotiate mutual monogamy or condom use [28]. Mathematical modeling of the CAPRISA 004 results showed that over the next two decades, tenofovir gel could prevent an estimated 1.3 million new HIV infections and over 800,000 deaths in South Africa alone [30]. These estimates do not take into account the added benefit of preventing HSV-2 infection that doubles the risk of acquiring HIV [31]. Thus, implemented on a broader scale, tenofovir gel could save millions of lives over time, thereby helping to ease the global burden of providing HIV treatment and care.

A few other recent studies have evaluated prophylactic use of antiretrovirals (ARVs) in women living in sub-Saharan Africa with disappointing results. The FEM-PrEP trial of daily oral Truvada (a combination of TDF and emtricitabine (FTC)) among women living in Uganda, South Africa, and Tanzania did not find a significant reduction in HIV incidence in the treatment arm compared to the placebo arm [32]. The Microbicide Trials Network VOICE trial, which tested daily dosing of tenofovir gel, oral TDF and oral TDF-FTC in women living in Uganda, South Africa, and Tanzania, also found no significant protective effect against HIV infection in any of the intervention groups [33]. Drug-level testing in FEM-PrEP and VOICE revealed very low adherence rates, which may have contributed to the lack of efficacy found in these trials [32, 33].

The difficulty in achieving high adherence to tenofovir gel or oral pre-exposure prophylaxis in trials of women in sub-Saharan Africa underscores the need for research into the implementation of pre-exposure prophylaxis in the region. The potential impact of a prophylactic intervention such as tenofovir gel could be severely hindered if it is not effectively implemented through the health system and made accessible to its target population. Thus, a good understanding of the strengths and challenges of local health services delivery systems prior to roll-out is imperative to the success of a new intervention.

One approach to programmatic scale-up of tenofovir gel within the public health system in South Africa is to integrate its provision into family planning services. Large numbers of sexually active women already utilize family planning services on a regular basis, and family planning staff are knowledgeable about sexual reproductive health and have experience providing counseling and adherence support. Additionally, family planning services are integrated into primary health care (PHC) services and are provided at no cost through the public sector health care delivery system, making these services widely available throughout South Africa. For these reasons, integrating HIV prevention and family planning services could be achieved with little additional burden to family planning providers and to the great benefit of their clientele.

Empiric evidence is needed to assess whether integrating tenofovir gel provision into family planning services can achieve similar, if not better, levels of safety and gel use than that observed in the CAPRISA 004 trial. CAPRISA 008 (protocol version 2.0, dated 23 November 2011) will rigorously test whether the quality improvement (QI) approach can be utilized in strengthening existing family planning services to the extent that they can incorporate tenofovir gel provision in their services and provide rapid, efficient, safe and effective access to a much needed product. Additionally, by including participants from CAPRISA 004 as participants in this study, a priority population will gain access to tenofovir gel and generate important ongoing data on the long-term safety of tenofovir gel.

## Methods

### Study design

This is a two-arm, open-label, randomized controlled non-inferiority trial.

### Study setting

This study will be conducted at the urban and rural CAPRISA Clinics that participated in the CAPRISA 004 study and their neighboring public sector PHC clinics where family planning services are provided in KwaZulu-Natal, South Africa. The CAPRISA Vulindlela Clinic, the rural control site for this study, is situated in Vulindlela, a rural sub-district with approximately 90,000 residents located in the KwaZulu-Natal midlands. The Vulindlela clinic adjoins the Mafakathini PHC clinic, which services an average of 500 clients per month seeking family planning services and will serve as the rural intervention site for CAPRISA 008.

The CAPRISA eThekwini Clinic, the urban control site for CAPRISA 008, and the Prince Cyril Zulu Communicable Disease Centre (PCZCDC) are situated in Warwick triangle, which is a public transportation hub for buses, “minibus” taxis and the commuter rail in Durban’s Central Business District. The PCZCDC, a PHC clinic of the Durban City Health Department, is primarily designated for the diagnosis and treatment of sexually transmitted infections (STIs) and tuberculosis but also offers family planning services to an average of 100 clients per month. It was planned that PCZCDC would serve as the urban intervention site for CAPRISA 008, but municipal approval to use the clinic for this purpose is still pending. In order to meet ethical obligations for post-trial gel access for CAPRISA 004 participants while awaiting municipal approval, a family planning clinic was created within the eThekwini clinic and will be utilized as the urban intervention site.

In order to facilitate the process of integrating tenofovir gel into existing family planning services during this study, a QI approach will be utilized to assist public sector family planning services with expanding their current service delivery to include tenofovir gel provision. The QI approach seeks to design systems for maximum effectiveness, efficiency, and adaptability and to disseminate the best models for health service delivery at the most rapid rate possible [34, 35]. Health care QI principles and the Model for Improvement provide an effective approach to help close the gap between evidence-based knowledge and the ability of health systems to implement large-scale programs [35, 36]. Health systems are strengthened using small scale, rapid cycles of improvement that are designed and implemented by local providers to develop reliable processes for service delivery through mentored coaching and support.

### Study population

The study will include a maximum of 700 women who previously participated in an ARV prevention study and are willing to use tenofovir gel through this open-label study at the CAPRISA Vulindlela and eThekwini Clinics and their neighboring primary care clinics.

#### HIV prevalence and incidence rates among source populations

Annual antenatal surveys conducted by CAPRISA have shown that the prevalence of HIV infection in pregnant women in Vulindlela has increased from 32.4% (95% CI, 27.5 to 37.3) in 2001 to 44.2% (95% CI, 39.4 to 49.0) in 2004. A pre-CAPRISA 004, prospective cohort study conducted from 2004 to 2005 that recruited women from the Mafakathini PHC clinic and the PCZCDC found a baseline HIV prevalence of 35.7% and 59.3%, respectively [37]. More recently, the CAPRISA 004 trial found an overall HIV incidence rate of 9.1 per 100 women years (95% CI, 6.9 to 11.7) at the urban and rural sites [28].

#### Eligibility criteria

Women aged 18 years and older who previously participated in an ARV prevention study are considered eligible if they are sexually active, HIV-negative, not pregnant, agree to use a non-barrier form of contraceptive for the duration of the trial, and are currently utilizing or agree to attend designated public sector family planning services.

Volunteers who have a creatinine clearance <50 ml/min, as estimated using the method of Cockcroft and Gault [38] or have any other condition that, based on the opinion of the Investigator or designee, would preclude provision of informed consent, make participation in the study unsafe, complicate interpretation of study outcome data, or otherwise interfere with achieving the study objectives will be excluded from the study.

#### Participant retention

The target retention rate is 90% per annum and participants will be tracked by the Protocol Team. Once a participant is enrolled in the study, study staff will make every reasonable effort to ensure adequate locator information is available for follow-up tracking. Participants will be tracked telephonically and/or by home visit, if necessary. Retention efforts will be conducted by the same team for both arms and have been standardized across study arms.

#### Participant withdrawal

Participants may voluntarily withdraw from the study for any reason at any time. Designated study staff may also withdraw participants from the study in order to protect their safety. Participants may also be withdrawn if the South African Medicines Control Council (MCC) or the University of KwaZulu-Natal’s (UKZN) Biomedical Research Ethics Committee (BREC) terminates the study prior to its planned end date.

#### Co-enrollment guidelines

Participants in this study may not take part in any other concurrent research studies that would interfere with the objectives of this study. The determination of whether participation in another study would be exclusionary for a given participant will be made by the Principal Investigators. Approved co-enrollment in other concurrent protocols will be documented.

### Study procedures

#### Informed consent

Informed consent is obtained from each study participant in English or isiZulu (the local African language) prior to screening and enrollment, in accordance with Good Clinical Practice guidelines. Consent for specimen storage is also sought. Participants are provided with copies of their informed consent forms if they are willing to receive them. An impartial witness is required for the entire informed consent process with any participant who is illiterate or whose literacy is limited. Documentation of the presence of a witness will be achieved through their signature on the informed consent document. Illiterate participants will indicate their consent via use of their mark (finger/thumb print) on the informed consent documents.

#### Recruitment, screening, and enrollment

Eligibility for the study is assessed in a step-wise manner at screening and enrollment. Potential participants are invited to screen for the study and asked to provide informed consent for screening. They receive pre-test counseling and two rapid HIV tests are performed. Post-test counseling is provided and those testing positive or indeterminate on at least one rapid test are referred to one of several HIV/AIDS treatment programs. If both HIV test results are negative, the potential participant is asked to provide demographic information, behavioral eligibility information and locator information and undergoes a blood draw for creatinine levels and urine pregnancy testing.

If potential participants remain eligible based on their creatinine clearance test results, they will undergo a physical and pelvic examination with genital specimen collection for storage. Women who meet all the study eligibility criteria will be asked to provide informed consent and are thereafter enrolled in the study. Blood will be drawn for hematology, liver function tests, blood chemistry tests, serology, hepatitis B virus assays and serum and plasma archive.

CAPRISA 008 is a post-trial access study and will maintain an open cohort for the duration of the study.

#### Randomization

Enrolled participants will be assigned at random to one of the two study arms in a 1:1 ratio. The randomization list used to assign individual study participants to one of the two arms will be generated by a randomization statistician who is not otherwise involved in the study. This statistician will use a randomly permuted block design, stratified by site. The randomization statistician will provide the site with sealed, opaque randomization envelopes, sequentially labeled by a unique participant identifier. These envelopes will be assigned in sequential order to eligible study participants. Study arm allocation will be concealed until after a participant is deemed eligible to participate in the study. This is an open label trial, and there will be no blinding after the envelope is opened. After completion of randomization procedures, participants who are randomized to the intervention arm will be escorted to the family planning clinic for adherence counseling and gel dispensation.

#### Study intervention

Participants randomized to the intervention arm will receive tenofovir gel through the Mafakathini PHC clinic in Vulindlela and the CAPRISA family planning clinic or PCZCDC in eThekwini. A QI approach will be used at the intervention sites to assist in the expansion of family planning services to include tenofovir gel provision. A QI advisor will work with the staff to conduct a gap analysis of existing family planning service provision prior to the enrollment of study participants at these sites. External and internal ideas will be carefully vetted to improve the quality of family planning service delivery. Once the initial QI process has been completed, a site initiation assessment will be undertaken to ensure procedures are in place for the study including procedures for dispensing tenofovir gel. This two-step approach of initially strengthening the family planning services and introducing the tenofovir gel using a QI framework will create a cadre of service providers who can remain vigilant about the quality of services provided and cope with unexpected or unanticipated situations. There will not be any additional QI effort in the control clinics beyond what is routinely done by the research clinics.

#### Follow-up visit procedures

Women randomized to the intervention arm will attend monthly visits at the family planning clinics for the first 3 months of study participation. Thereafter, gel provision and monitoring will be scheduled to coincide with each participant’s routinely scheduled family planning visits (typically every 2 to 3 months). Follow-up visits for women enrolled into the control arm are scheduled throughout the study follow-up period on a 28-day schedule. The visit window around a study visit is 14 days on either side.

Upon enrollment and at each study visit, adherence counseling will be provided to study participants when they receive gel supplies. The CAPRISA 004 before and after dosing strategy will be utilized in the intervention and control arms of this study. Participants will be advised to insert the first gel dose up to 12 hours ***b***efore sex, insert the second gel dose as soon as possible within 12 hours ***a***fter sex, and not to insert more than **t**wo gel doses in a ***24*** hour period (referred to as BAT 24). Adherence counselors will utilize techniques based on motivational interviewing to address participant-centered strategies to remember proper dosing, to ensure availability of gel when away from home and to identify and discuss various challenges and situations that may impede product use. Counseling will include reminders to contact study staff with questions about gel use and requests for additional gel supplies.

At each scheduled study visit, enrolled participants in the intervention and control arm will also be provided with the following: HIV risk reduction counseling and male and female condoms; contraception counseling and provision of contraceptive method of choice; advice to contact study staff with questions about the study, with requests for additional counseling, additional condoms and gel or contraception, and/or to report adverse events (AEs).

In addition to the regular follow-up requirements, additional procedures, including a pelvic examination, genital specimen collection, and scheduled blood draws for STI testing and plasma and serum archive, will be completed at study months 6, 12, 18, 24, study exit and when clinically indicated.

#### Laboratory procedures

Serum, plasma, and genital specimens taken at enrollment and designated follow-up visits will be stored for assessments of markers of safety, risk exposure, product adherence, activity against STIs, and tenofovir resistance. In addition, stored plasma will be used for retrospective ribonucleic acid polymerase chain reaction or Western blot testing to confirm whether early incident cases of HIV infection during the trial occurred post-randomization.

### Study product considerations

#### 1% Tenofovir gel

Tenofovir gel is a clear, transparent, viscous gel at a concentration of 1% (weight per weight). For the purposes of this study, the gel will be packaged in single-use, individually wrapped and labeled pre-filled opaque applicators containing approximately 4 ml of gel.

#### Adherence assessment

Data on adherence to the gel use regimen will be collected at each study visit via brief interviewer-administered instruments. Study participants will also be asked to return all previously dispensed applicators at each visit. Additionally, genital specimens collected during the trial at study months 6, 12, 18, 24, study exit and from suspected HIV seroconverters will be archived for analysis of markers of product adherence to enhance interpretation of the results of the trial.

#### Discontinuation of product

Study participants will be discontinued from using tenofovir gel by the Principal Investigators and their designees in the event that they experience a serious adverse event (SAE) that is judged by the study clinician or designee to be related to gel use. Participants who become pregnant will temporarily discontinue gel use and will only resume gel use when their pregnancy test reverts to negative. The Principal Investigators and designees also may at their discretion discontinue gel use - temporarily or permanently - among participants who experience an AE judged to be related to gel use, have a pelvic examination finding involving deep epithelial disruption that is not resolving, are unable or unwilling to comply with study procedures, or otherwise might be put at undue risk to their safety and well-being by continuing gel use. Gel use will be permanently discontinued when HIV infection has been confirmed.

#### Concomitant medications

Enrolled study participants may continue use of all concomitant medications, including prescription, non-prescription, traditional, and other preparations during this study. Participants will be encouraged to avoid douching and the use of vaginally-applied medications/preparations. All concomitant medications used by participants throughout the course of the study will be reported on applicable case report forms (CRFs).

### Safety monitoring and adverse event reporting

#### Adverse events and reporting requirements

An AE is defined as any untoward medical or social occurrence in a clinical research participant which may or may not have a causal relationship with tenofovir gel. Study participants will be provided with contact telephone numbers and instructed to contact study staff to report any AEs they may experience, except for life-threatening events for which they will be instructed to seek immediate emergency care. All participants reporting an AE will be followed clinically until the AE resolves or stabilizes. AEs that are ongoing at the time of study exit will be followed up for up to 30 days after study exit and then, if not resolved, will be referred to a health care provider for further follow-up.

For each AE, an assessment of the relatedness to the study product will be made using the criteria and scale as outlined in the study specific procedures manual and graded for severity using the most current National Institutes for Health Division of AIDS severity grading system. All AEs will be reported on the appropriate CRF and will contain at least the date the AE occurred, a brief description of the event, the relationship to study product, the action taken, the outcome, date resolved, and the seriousness of the event.

#### Serious adverse event reporting

A SAE includes any experience that is fatal or life-threatening, results in persistent or significant disability/incapacity, requires or prolongs hospitalization, or is a congenital anomaly. A life-threatening AE means that the participant was, in the view of the designated study staff, at immediate risk of death from the condition as it occurred. All SAEs will be reported on the appropriate CRF and, with permission of the participant, records from all non-study medical providers related to SAEs will be obtained and required data elements will be recorded on study CRFs. Notification of deaths will be recorded by reflecting the medical condition that led to the death on the appropriate CRF. Reporting SAEs may require additional detailed reports and follow-up. All SAEs will be reported to CONRAD, the South African MCC and the UKZN BREC.

#### Data and safety monitoring

The study statisticians will prepare routine study progress reports which include reports of AEs experienced by study participants for review by the Data and Safety Monitoring Board (DSMB). DSMB members will meet in person and/or via teleconference throughout the period of study implementation to conduct interim reviews of study progress across both arms, including rates of participant accrual, retention, completion of primary and relevant secondary endpoint assessments, and clinical and laboratory adverse events including the SAE rate. Any deaths of study participants or other SAEs will be reviewed and the final decision taken by the DSMB documented. Following review of the data during the trial, the DSMB may recommend that the study proceed as designed, proceed with design modifications, or be discontinued.

### Statistical considerations

#### Primary and secondary endpoints

The primary endpoint for this study is mean gel use, assessed by the number of returned used applicators per participant per month. This endpoint was chosen because the CAPRISA 004 trial demonstrated a correlation between the number of returned used applicators and the level of effectiveness against HIV infection. The following secondary endpoints will be assessed: clinical and laboratory adverse event and pregnancy rates; HIV incidence rates; self-reported adherence to the tenofovir gel dosing strategy; self-reported service acceptability and completion rates of quarterly HIV and pregnancy testing; HIV viral load among HIV seroconverters; tenofovir resistance among HIV seroconverters; vaginal cytokines; HSV-2 and human papilloma virus incidence rates; tenofovir levels (and their correlation with HIV and HSV-2 infection rates); self-reported product acceptability.

#### Power and sample size

A maximum of 700 women, randomized in a 1:1 ratio, will be enrolled over approximately 12 months. We would like to demonstrate that the difference in gel use is no greater than 20% between the two study arms. Since the mean number of returned used applicators in CAPRISA 004 was five per participant per month, a 20% difference in gel use equates to one used applicator per participant per month. A maximum sample size of 700 provides >90% power to demonstrate whether gel use in women attending family planning services is similar to, but no more than 20% lower, than gel use by women attending the CAPRISA research clinics, adjusted for 10% loss to follow-up.

#### Data analysis

The primary objective of this non-inferiority study is to demonstrate that gel use, as measured by the mean number of returned used applicators per participant per month, in the family planning arm is not more than 20% lower than the CAPRISA clinic arm. The number of used applicators returned will be compared between the two treatment arms using an appropriate longitudinal method, such as generalized estimating equations or linear mixed models, adjusted for site. A one-sided test will be used to determine whether the difference in mean gel use between the intervention arm and the control arm is less than 20%.

Analyses will be performed on an intention-to-treat (ITT) basis and on the per-protocol and as-treated populations. For the ITT analysis, participants will be analyzed according to the study arm, even if the participant did not follow the assigned procedures. Because the ITT analysis could increase the chances of declaring equivalence in a non-inferiority study, per-protocol analyses will also be conducted and compared to the ITT analyses before the decision is made to regard the ITT population as the primary population.

#### Data management

Data will be collected on standardized CRFs, which will be developed by the study team. Source documents will be maintained in the participant’s medical chart or study file at the site. CRFs will be faxed to the central CAPRISA Data Management Server using the DataFax system (Clinical DataFax Systems Inc., Ontario, Canada). Queries arising during validation of the data will be recorded in quality control reports sent to the sites on a regular basis. The original CRFs and related documents will be stored securely at the sites, both during and after the completion of the study.

### Ethical considerations

#### Regulatory and ethical review

This study is being conducted under the regulatory oversight of the South African MCC (reference number: 20110145) and the ethical oversight of UKZN’s BREC (reference number: BFC 237/010) and in accordance with International Conference on Harmonization guidance and Good Clinical Practice standards. The study was initiated after receipt of approval by the MCC (approval date: 21 May 2012) and UKZN’s BREC (approval date: 13 September 2012). The study is being conducted in accordance with all conditions of approval by the ethics and regulatory committees.

#### Community involvement and consultation

The CAPRISA Community Program has, through a consultative process, established CAPRISA Community Research Support Groups at both the CAPRISA sites where this study will be conducted. The membership of these Community Research Support Groups includes local community leaders, traditional leaders, leadership of local HIV/AIDS organizations, previous study participants, local health service provider representatives and HIV-positive local community members. In preparation for this trial, the CAPRISA Community Program will inform, educate and mobilize the community to enhance community input into the research process.

#### Confidentiality

Every effort will be made to protect participant privacy and confidentiality to the extent permitted by law.

## Discussion

CAPRISA 004 established proof-of-concept that tenofovir gel is safe and effective in preventing HIV infection in a rigorous clinical trial. Translating these findings into health service programs poses many challenges that can be exacerbated in the context of weak health care delivery systems. The post-trial, pre-licensure window is a critical period to prepare and devise effective strategies for informing future policy and programmatic scale-up of new interventions. CAPRISA 008 will answer important implementation questions about how best to incorporate tenofovir gel into routine family planning health services in South Africa and how to make it accessible to women who would benefit most from this product. By undertaking this study now, prior to licensure, stakeholders will be better prepared for widespread roll-out and scaling up of provision of tenofovir gel within the public health system of South Africa.

This study will also add to the growing body of implementation research for HIV prevention. Implementation science uses the scientific method to investigate how to integrate research findings and evidence-based interventions into health care policy and practice [39]. Several recent studies have generated useful data for guiding the implementation of programs to reduce mother-to-child transmission of HIV and increase uptake of medical male circumcision for HIV prevention [40–43]. However, CAPRISA 008 is the first study to evaluate implementation strategies for an HIV prevention tool that can be used by women to protect themselves from infection over a long period of time.

There is increasing consensus that research participants in developing countries should have post-trial access to beneficial interventions based on the ethical principles of beneficence and justice [44]. During the waiting period for confirmatory studies and the licensure of tenofovir gel, CAPRISA has a responsibility to make the gel available to CAPRISA 004 participants since it is unavailable to them through any other channel. By inviting CAPRISA 004 participants to take part in the CAPRISA 008 study, we are able to meet this post-trial ethical obligation whilst generating important data for the future implementation of tenofovir gel within the health service.

## Trial status

Participant recruitment, screening and enrollment are ongoing.

## Abbreviations

AE: adverse event
ARV: antiretroviral
BREC: Biomedical Research Ethics Committee
CAPRISA: Centre for the AIDS Programme of Research in South Africa
CRF: case report form
DSMB: Data and Safety Monitoring Board
FTC: emtricitabine
HSV-2: herpes simplex virus type 2
ITT: intention-to-treat
MCC: Medicines Control Council
PCZCDC: Prince Cyril Zulu Communicable Disease Centre
PHC: primary health care
QI: quality improvement
SAE: serious adverse event
STI: sexually transmitted infection
TDF: tenofovir disoproxil fumarate
UKZN: University of KwaZulu-Natal.
